# Glucotoxicity induces abnormal glucagon secretion through impaired insulin signaling in InR1G cells

**DOI:** 10.1371/journal.pone.0176271

**Published:** 2017-04-20

**Authors:** Takashi Katsura, Dan Kawamori, Eri Aida, Taka-aki Matsuoka, Iichiro Shimomura

**Affiliations:** Department of Metabolic Medicine, Graduate School of Medicine, Osaka University, Osaka, Japan; International University of Health and Welfare School of Medicine, JAPAN

## Abstract

The significance of glucagon in the pathophysiology of diabetes mellitus is widely recognized, but the mechanisms underlying dysregulated glucagon secretion are still unclear. Here, we explored the molecular mechanisms of glucagon dysregulation, using an *in vitro* model. Hamster-derived glucagon-secreting InR1G cells were exposed to high glucose (25 mM) levels for 12 h before analyzing glucagon secretion and the activity of components involved in insulin signaling. High-glucose treatment induced increased glucagon secretion in InR1G cells, which represents a hallmark of diabetes mellitus. This treatment reduced the phosphorylation of Akt, indicating the deterioration of insulin signaling. Simultaneously, oxidative stress and JNK activity were shown to be increased. The inhibition of JNK signaling resulted in the amelioration of high-glucose level-induced glucagon secretion. Abnormally elevated glucagon secretion in diabetes can be reproduced by high-glucose treatment of InR1G cells, and the involvement of high glucose-oxidative stress-JNK-insulin signaling pathway axis has been demonstrated. These data elucidate, at least partly, the previously unclear mechanism of abnormal glucagon secretion, providing insights into a potential novel approach to diabetes treatment, targeting glucagon.

## Introduction

The pathophysiological significance of glucagon is increasingly recognized, and glucagon is considered a potential new therapeutic target for diabetes mellitus treatment, since its dysregulated secretion in the diabetic state affects glycemic status [1]. In diabetes, glucagon secretion is paradoxically increased, contributing to the exacerbation of the already existing hyperglycemia. In contrast to this, defective glucagon response in hypoglycemic states exacerbates clinical symptoms of hypoglycemia. A recent report demonstrated that the suppression of glucagon secretion and the enhancement of insulin secretion equally contribute to the glucose-lowering properties of glucagon-like peptide (GLP)-1 [2]. It is generally recognized that glucagon secretion is regulated by systemic glycemic status [3], although the mechanisms underlying this regulation remain unclear. However, the regulatory mechanisms of glucagon secretion dependent on various nutrients, the central and autonomic nervous system, and the endocrine system, including incretins and somatostatin, are well known [4–6]. In addition to these classical glucagon regulators, we have demonstrated that insulin signaling in α-cells represents one of the central physiological regulators of glucagon secretion *in vivo* [7, 8].

Insulin produced by β-cells has been proposed as one of the intra-islet paracrine factors that can modulate the secretion of glucagon from neighboring α-cells. Furthermore, various components of the insulin signaling pathway, including the insulin receptor, are abundantly expressed in α-cells, suggesting an important role of insulin signaling in these cells [9–11]. Recent *in vitro* studies using α-cell lines demonstrated a role of the insulin signaling pathway in the suppression of glucose-induced glucagon secretion [12], as well as in the stimulation of glucagon secretion by low concentrations of glucose [13]. Insulin signaling was reported to suppress glucagon secretion in two different manners: by reducing the sensitivity of K^+^_ATP_ channels via phosphatidylinositol 3-kinase (PI3K) [11, 14] and by enhancing GABA receptor recruitment via Akt [15, 16].

Despite extensive investigations of various physiological regulatory mechanisms of glucagon secretion, the mechanism(s) underlying abnormal glucagon secretion in diabetes is still not completely elucidated [17]. In this study, we focused on the insulin signaling, shown to be an important endogenous mechanism of physiological regulation of glucagon secretion in α-cells [8]. We explored potential molecular mechanisms underlying dysregulated glucagon secretion, using a glucagon-secreting InR1G cells, and showed that high-glucose treatment induces abnormally elevated glucagon secretion in these cells through impaired insulin signaling.

## Materials and methods

### Islet isolation and secretion

Pancreatic islets were obtained from 8-week-old male C57B6 mice, using collagenase (Librase; Roche, Switzerland) digestion [18]. After digestion, healthy round islets were hand-picked under a stereoscopic microscope, then incubated for 12 hours in RPMI1640 medium (Nacalai Tesque, Japan) containing 7 mM glucose supplemented with 10% v/v fetal bovine serum (FBS; Invitrogen/Thermo Fisher Scientific, USA). Twenty-four hours before the secretion experiments, the islets were divided into 2 groups and placed in RPMI media containing different concentration of glucose (7 or 15 mM) and supplemented with 10% v/v FBS. For the assessment of glucagon and insulin secretion, batches of 20 healthy size-matched islets were preincubated for 30 min in HEPES-balanced Krebs-Ringer bicarbonate (KRB) buffer containing 0.1% bovine serum albumin (BSA; A-7888, Sigma-Aldrich, U.S.A.) and 1 mM glucose. Subsequently, the islets were incubated for 60 min in KRB buffer containing 0.1% BSA with different glucose concentrations (1, 7, or 25 mM). The supernatants of the incubation buffers were used for the insulin and glucagon assays. Glucagon concentrations were measured using specific ELISA for glucagon (Mercodia, Sweden) immediately after the experiments, and insulin concentrations were measured by ELISA for insulin (Morinaga, Japan).

### Cell culture and treatment

Hamster glucagon-secreting InR1G cells (a kind gift from Dr. J. Philippe, University of Geneva, Switzerland) were cultured at 37°C in RPMI 1640 medium containing 11.1 mM of glucose, supplemented with 10% v/v FBS, 100 U/mL penicillin, and 100 μg/mL streptomycin, as previously described [19]. All experiments were performed using cells between passages 12 and 33. InR1G cells were replated 4 days before the experiments, and incubated in a regular growth medium as described above. Twelve hours before the experiments, cells were incubated in different stimulation media (RPMI 1640 with 11.1 or 25 mM of glucose without FBS) supplemented with/without 50 μM hydrogen peroxide (H_2_O_2_), 13.9 mM 2-deoxyglucose (2-DG), DMSO (0.1% v/v), and/or 10 μM SP600125 (Wako Pure Chemical Industries, Japan) and A6730 (Sigma-Aldrich, U.S.A.), and harvested for glucagon secretion and content, or cell number, and protein assays. Following the stimulation of 11.1 mM or 25 mM glucose, cells were stimulated with insulin (100 nM) or H_2_O_2_ (100μM) for 0, 5, or 15 min, and harvested for protein expression assays. For RNA expression assays, the cells were incubated for 12 or 36 h in RPMI media with 11.1 or 25 mM glucose without FBS. The cells were also incubated for 72 or 120 h in RPMI media supplemented with 10% v/v FBS, and 11.1 or 25 mM glucose; the media were changed 24 and 72 h after seeding.

### Glucagon secretion and content determination

Glucagon secretion from InR1G cells was assessed by static incubation [20]. Cells were settled in HEPES-balanced Krebs-Ringer bicarbonate (KRB) buffer supplemented with 1 mM glucose for 30 min, and afterward, incubated in stimulation KRB buffer, supplemented with different concentrations of glucose (1, 7, or 25 mM) with/without 10 mM L-arginine (L-Arg) for 60 min. Cell numbers were determined using TC20 automated cell counter (Bio-Rad, USA). Cellular glucagon content was assessed after acid-ethanol extraction (70% v/v ethanol supplemented with 0.18 N HCl).

### Assessment of gene expression

Total RNA was extracted from InR1G cells using RNeasy Mini Kit (Qiagen, Germany). cDNA samples were generated by using Verso cDNA Synthesis Kit (Thermo Fisher Scientific, USA). Preproglucagon gene expression was determined by quantitative real-time PCR using SYBR Green PCR Master Mix (Applied Biosystems/Thermo Fisher Scientific, USA), and the expression levels were normalized using the expression levels of 28S ribosomal RNA (rRNA) [21]. The following primers were used: glucagon, 5′-GATCATTCCCAGCTTCCCAG-3′ (forward), 5′-CTGGTAAAGGTCCCTTCAGC-3′ (reverse); 28S rRNA, 5′-TAGCCAAATGCCTCGTCATC-3′ (forward), 5′-ACCTCTCATGTCTCTTCACC-3′ (reverse) [22].

### Western blotting

For western blotting, 20 μg of cellular protein extracts were used [23]. We used rabbit anti-Akt antibody, anti-S473- and anti-T308-phospho-specific Akt antibodies, anti-PDK1 antibody, anti-S241-phospho-specific PDK1 antibody, anti-JNK antibody, anti-T183/Y185-phospho-specific JNK antibody, anti-p38 antibody, anti-phospho-specific p38 antibody (Cell Signaling Technology, USA), and anti-rabbit IgG horseradish peroxidase-conjugated secondary antibody (Santa Cruz Biotechnology, USA). Relative protein amounts were evaluated using Chemidoc XRS system (Bio-Rad, USA) and ImageJ tool (National Institute of Health, USA).

### Determination of glucagon, protein, and reactive oxygen species (ROS) levels

Glucagon levels were measured using glucagon-specific ELISA kit (Mercodia, Sweden), following the manufacturer’s instructions. Protein content was measured by BCA protein assay (Pierce/Thermo Fisher Scientific, USA). The levels of ROS were evaluated by OxiSelect ROS Assay (Cell Biolabs, USA).

### Recombinant adenovirus

Recombinant adenoviruses expressing the dominant-negative (DN) form of JNK and control adenoviruses, carrying green fluorescent protein (Ad-GFP) vector, were prepared using the AdEasy system as previously described [23]. Adenovirus titers were increased up to 1 × 10^10^ PFU/mL using the ViraKit adenovirus standard purification kit (Virapur, USA). The cells were infected with different adenoviral particles at the concentration of 3 × 10^8^ PFU/mL. Subsequent experiments were conducted 36 h after the initial addition of the viruses to the cultures. The efficiency of adenovirus-mediated gene transfers was determined to be between 50% and 70%, as shown by GFP expression.

### Statistical analysis

All data were analyzed using an unpaired two-tailed Student’s t-test or analysis of variance (ANOVA) with Tukey-Kramer’s post-hoc test, and are presented as mean ± standard error of the mean (SEM), and *P* value less than 0.05 was considered significant.

## Results

### High glucose treatment induces abnormal glucagon secretion in mouse isolated islets and InR1G cells

**Fig 1:**
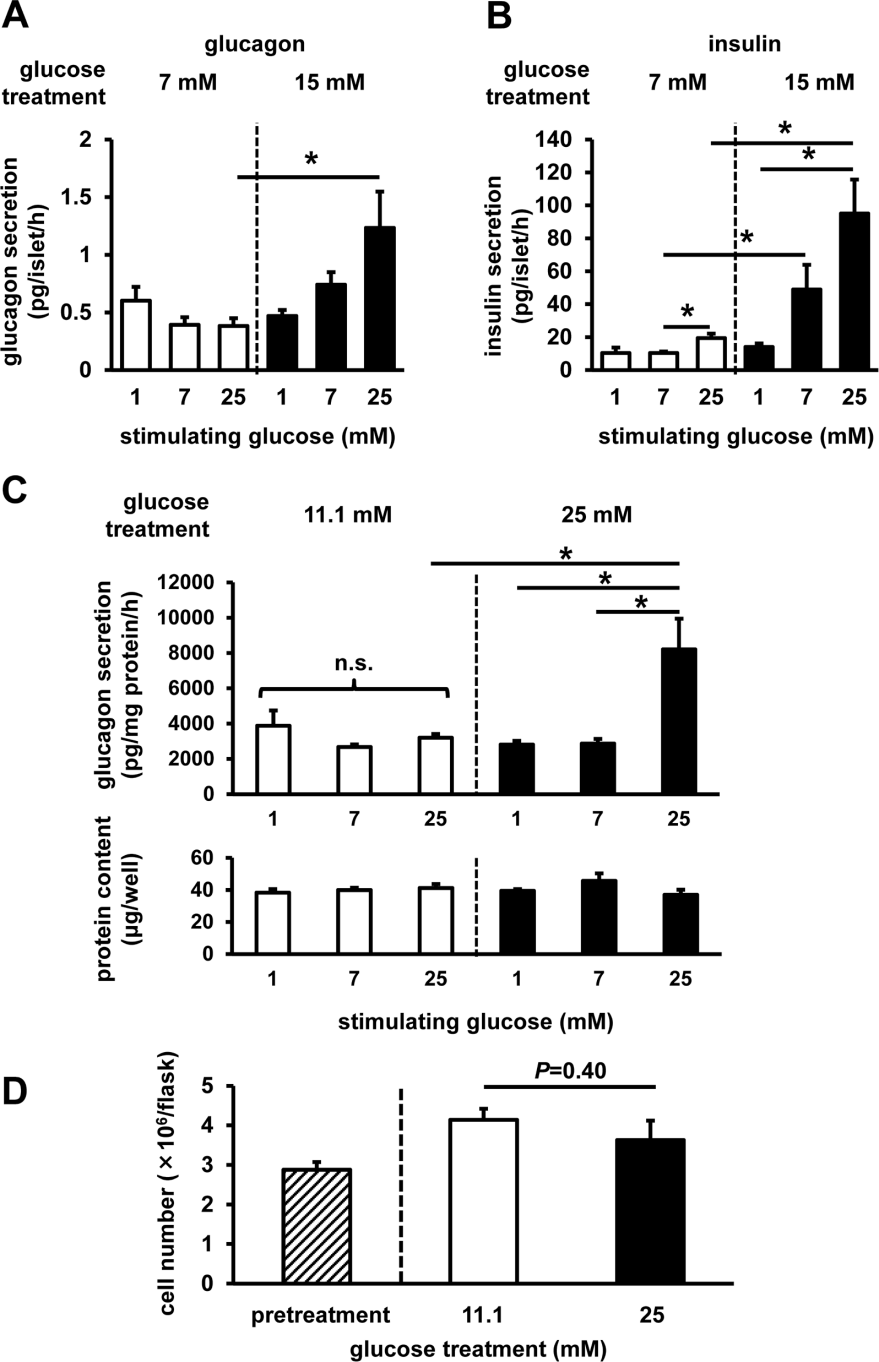
Glucagon secretion from mouse-isolated islets and InR1G cells. Islets were isolated from C57B6 mice, and then batches of 20 islets were subjected to static incubation experiments for glucagon (**A**) and insulin (**B**) secretion after pretreatment with 7 mM (white) or 15 mM (black) glucose (n = 3–4, each group). InR1G cells were pretreated with 11.1 mM (white) or 25 mM (black) glucose. (**C**) InR1G cell glucagon secretion (top) and protein content (bottom) (n = 3–4, each group). (**D**) Number of the InR1G cells pre- (hatched) and post-treatment (n = 3–4, each group). Data are expressed as mean ± SEM; *P<0.05, between the indicated groups.

First, to investigate the possible impact of high glucose load on glucagon secretion, we assessed the hormone secretion from mouse-isolated islets. Under regular culture conditions with 7 mM glucose which is similar to physiological blood glucose levels, glucagon secretion was not elevated after 25 mM glucose stimulation, whereas insulin secretion was significantly increased (**Fig 1A and 1B**). In contrast, after high-glucose-level treatment (15 mM glucose for 24 h, mimicking diabetic hyperglycemia), glucagon secretion was significantly elevated after 25 mM glucose stimulation (**Fig 1A**). Insulin secretion, which can affect glucagon secretion from neighboring α-cells, also significantly increased after 7 and 25 mM glucose stimulation (**Fig 1B**).

Next, to directly assess the cellular intrinsic mechanism(s) underlying high glucose-induced increase in glucagon secretion, we evaluated the glucagon secretion from glucagon-secreting InR1G cells. Under regular culture conditions (11.1 mM glucose), glucagon secretion following the stimulation of cells with 25 mM glucose was comparable to that in the cells stimulated with different levels of glucose (**Fig 1C**). After high-glucose-level treatment (25 mM glucose, 12 h), glucagon secretion exhibited a considerable increase after 25 mM glucose stimulation, whereas glucagon levels did not significantly change after 1 mM and 7 mM glucose stimulation (**Fig 1C**). Cellular protein content after the static incubation (**Fig 1C**) and cell numbers (**Fig 1D**) were not altered after 12 h of high-glucose treatment.

**Fig 2:**
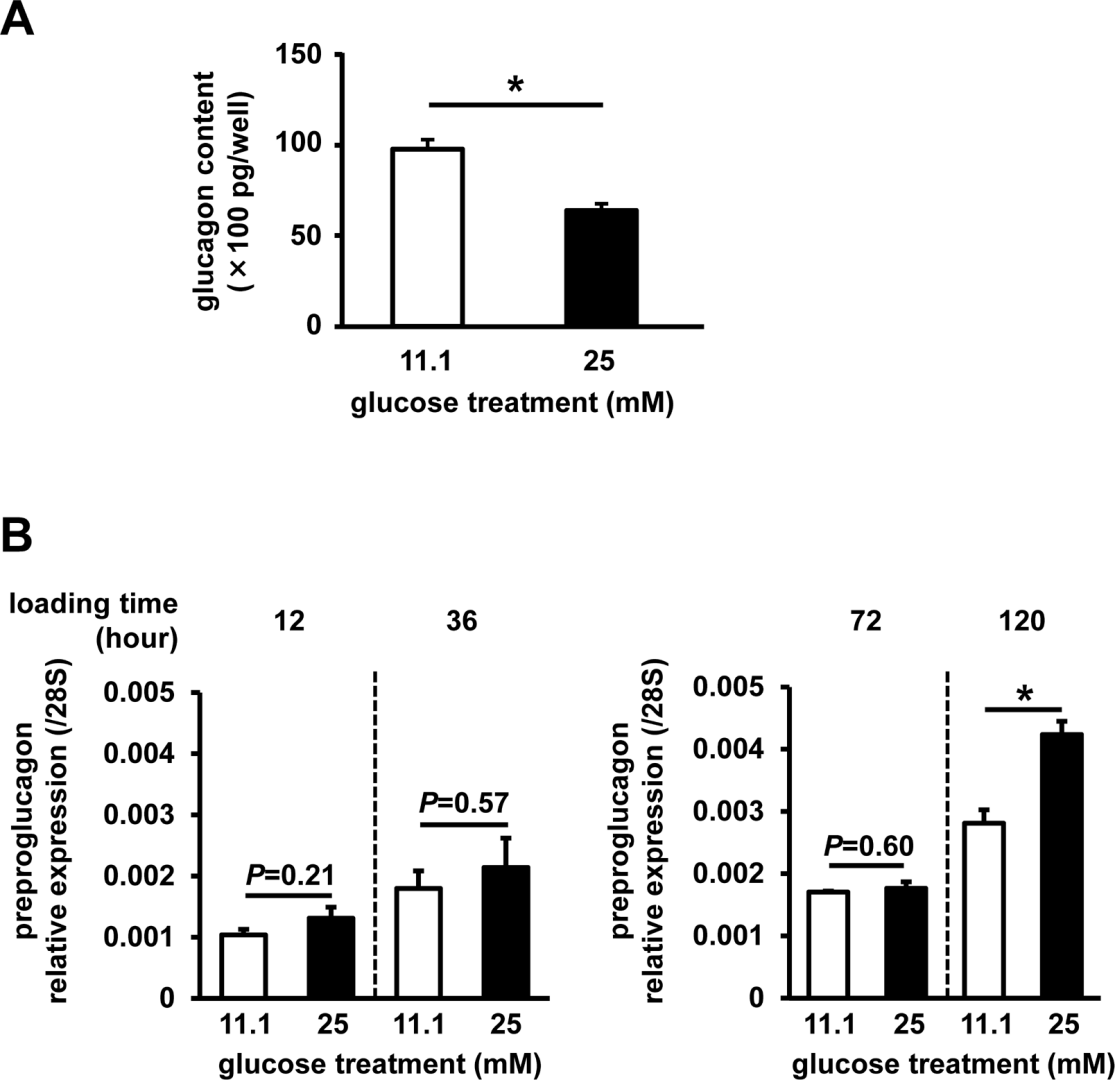
Glucagon content and preproglucagon expression. Cells were pretreated with 11.1 mM (white) or 25 mM (black) glucose. (**A**) Glucagon content following static incubation (n = 3–4, each group). (**B**) Preproglucagon expression (n = 6, each group). Data are expressed as mean ± SEM; *P<0.05, between the indicated groups.

High-glucose treatment to InR1G cells led to a significant decrease in cellular glucagon content (**Fig 2A**). In contrast, gene expression of preproglucagon was shown to increase after 12, 36, and 72 h of high-glucose treatment, but this increase was not statistically significant (**Fig 2B**). The upregulation became significant after 120 h of high-glucose treatment, as reported previously [24].

### High glucose exposure impairs Akt phosphorylation

**Fig 3:**
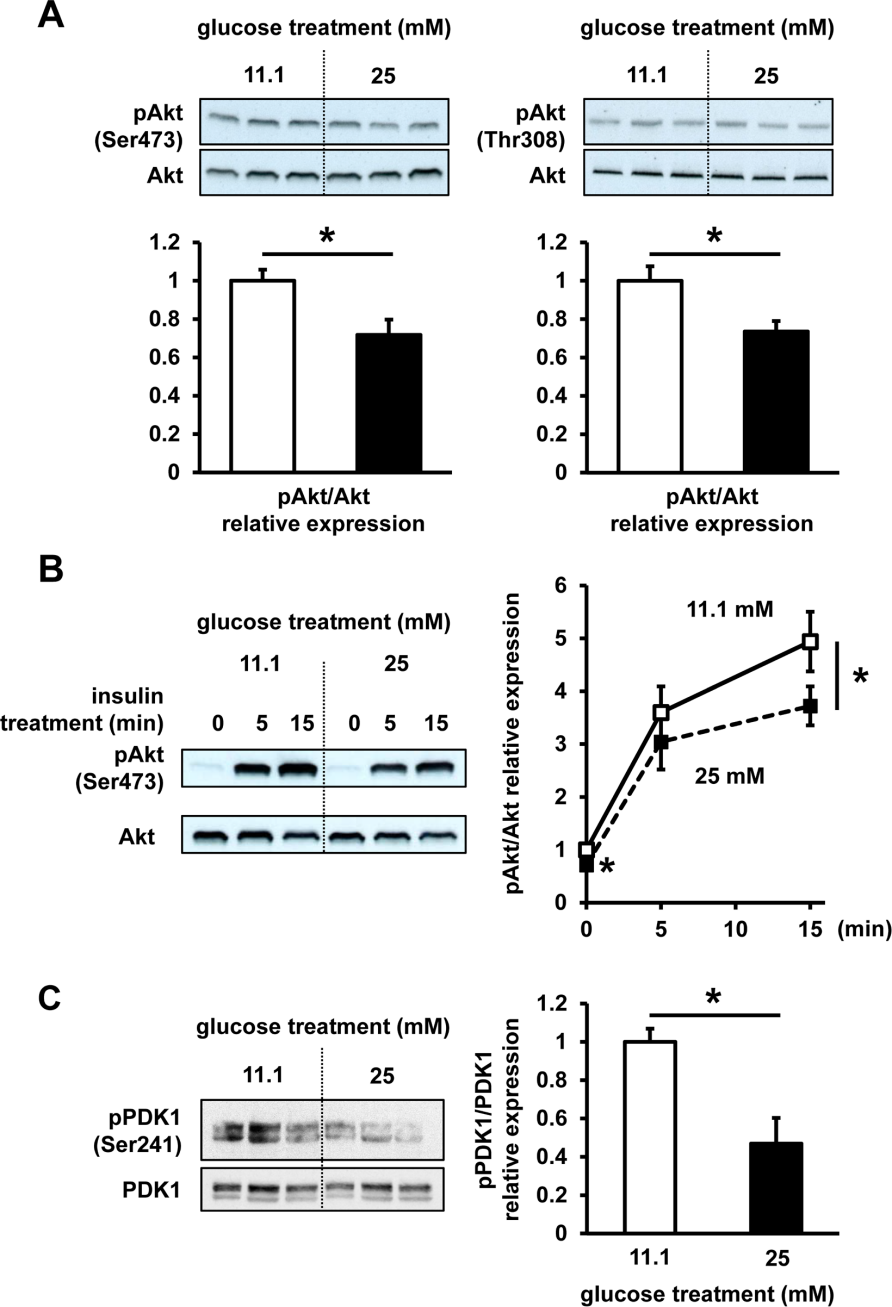
Akt and PDK1 phosphorylation status. InR1G cells were exposed to regular (11.1 mM, white) or high (25 mM, black) glucose levels for 12 h. (**A**) Ser473 and Thr308 phospho-(p)Akt/Akt ratios were determined. (**B**) Following the stimulation of 11.1 mM or 25 mM glucose-treated cells with insulin (100 nM) for 0, 5, or 15 min, the ratio of Ser473 pAkt/Akt was determined. Representative images selected from 3 independent experiments are shown. (**C**) Phospho-(p)PDK1/PDK1 ratios were determined. Relative expression of pAkt and pPDK1 was determined using densitometry and normalized using total Akt and PDK1 levels. n = 3–4 in each group. Data are expressed as mean ± SEM; *P<0.05, between the indicated groups.

To assess a possible impact of chronic high-glucose treatment on insulin signaling, Akt phosphorylation status, one of the central components of this signaling, was evaluated in InR1G cells. Following the high-glucose treatment, baseline phosphorylation status of Akt was significantly reduced, by 30% at Ser473 residue and by 25% at Thr308 residue, compared with that at regular culture conditions (**Fig 3A**). Additionally, a similar 20% impairment of phosphorylation at Akt Ser473 was observed at 15 min after insulin stimulation (**Fig 3B**), while the phosphorylation status of PDK1, an upstream Akt kinase, was reduced after the high-glucose treatment (**Fig 3C**).

### High glucose load induces oxidative stress and JNK activation

**Fig 4:**
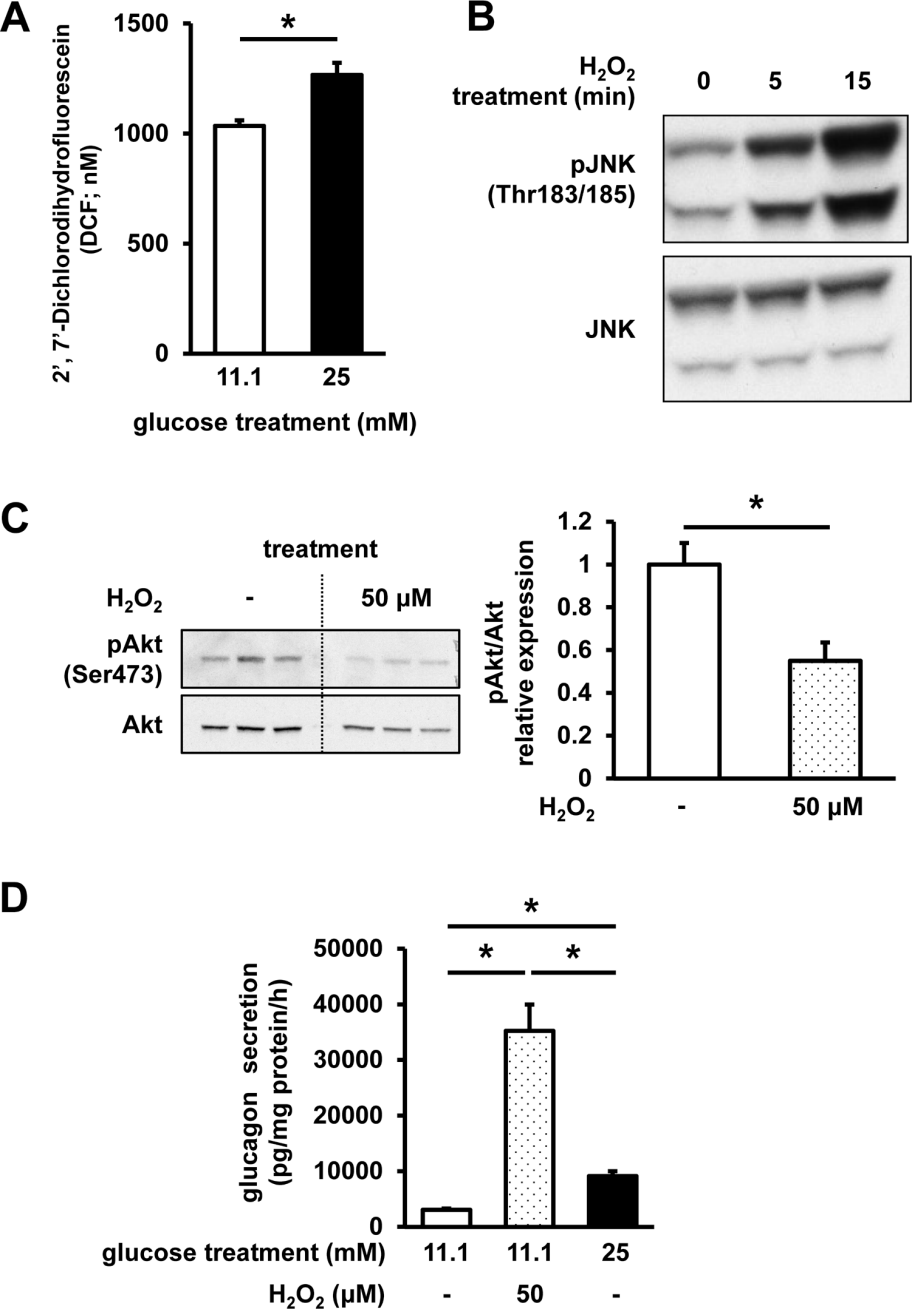
ROS content and the effects of oxidative stress on InR1G cells. (**A**) ROS content determination. Cells were exposed to regular (11.1 mM, white) or high (25 mM, black) glucose levels for 12 h; n = 7; data are expressed as mean ± SEM; *P<0.05, between the indicated groups. (**B**) JNK phosphorylation levels. InR1G cells were exposed to 11.1 mM glucose for 12 h, and stimulated using H_2_O_2_ (100 μM) for 5 or 15 min. (**C**) Akt phosphorylation and (**D**) glucagon secretion levels following the exposure of the cells to regular (11.1 mM) glucose levels in combination with (dotted)/without (white) 50 μM H_2_O_2_, or high (25 mM, black) glucose levels for 12 h, and the glucagon secretion was assessed after the stimulation with 25 mM glucose. Relative pAkt expression was determined using densitometry and normalized using total Akt levels; n = 4, each group. Data are expressed as mean ± SEM; *P<0.05, between the indicated groups.

Next, to elucidate the mechanisms underlying the impaired glucagon secretion, we assessed the possible involvement of oxidative stress. ROS content was shown to be significantly increased in the cells cultured in high-glucose medium, compared with that of the cells grown under regular glucose (11.1 mM) conditions (**Fig 4A**). Furthermore, we confirmed that the induction of oxidative stress in InR1G cells, using hydrogen peroxide (100 μM, 15 min), significantly induces the phosphorylation of JNK, an important stress-signal transducing kinase (**Fig 4B**). In order to examine the effects of oxidative stress on insulin signaling in InR1G cells, oxidative stress was induced by the treatment with 50 μM H_2_O_2_ for 12 h, which was shown to significantly reduce Akt phosphorylation levels at Ser473 residue by 40% (**Fig 4C**). Additionally, H_2_O_2_ treatment induced a large increase in glucagon secretion (**Fig 4D**).

**Fig 5:**
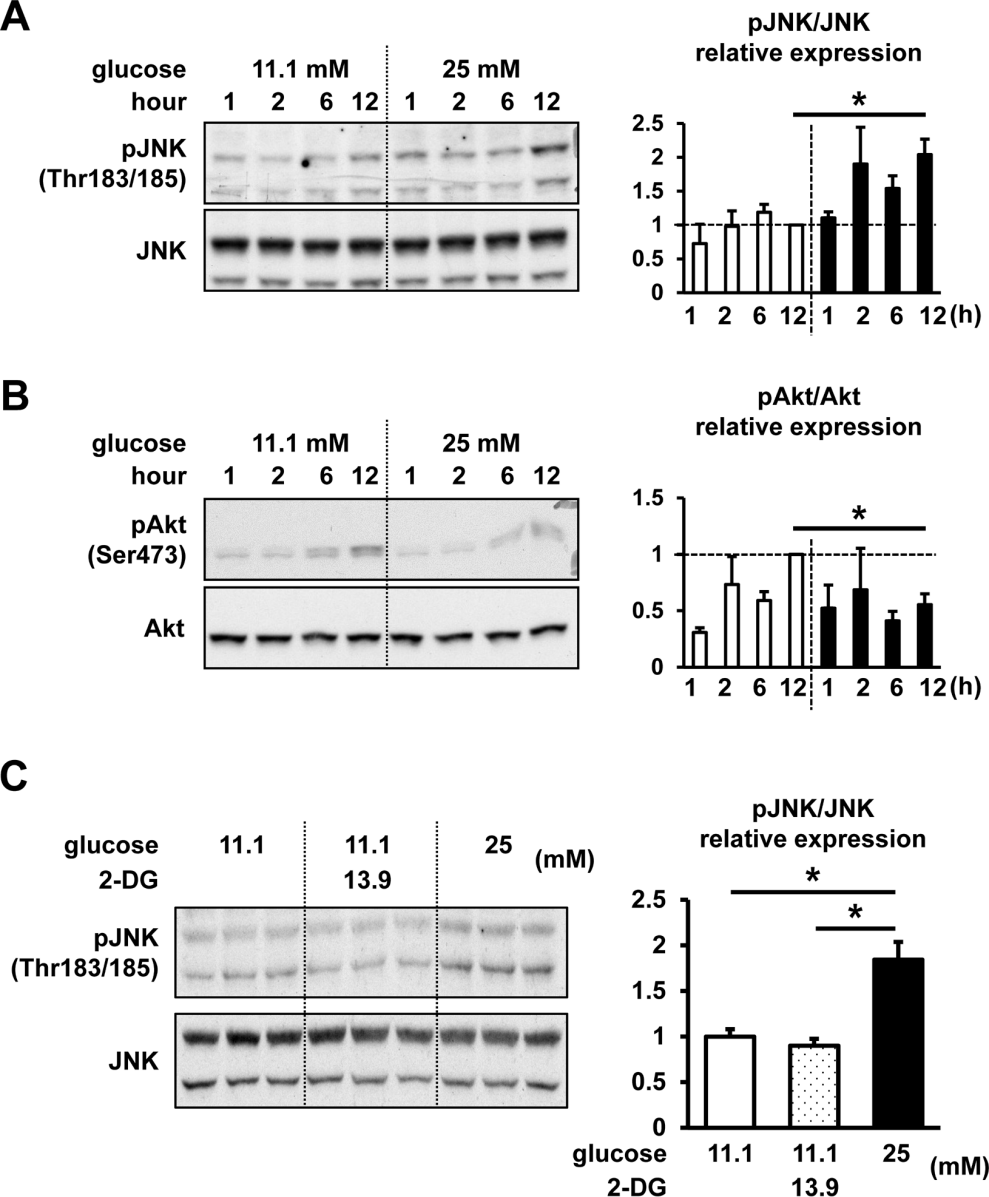
Time-dependent effects of different glucose concentrations on Akt and JNK phosphorylation levels. InR1G cells were treated with regular (11.1 mM, white) or high (25 mM, black) glucose levels for 1, 2, 6, and 12 h. (**A**) JNK phosphorylation levels. (**B**) Akt phosphorylation levels. (**C**) InR1G cells were exposed to regular (11.1 mM) glucose concentration, in combination with (dotted) or without (white) 13.9 mM 2-DG, or high (25 mM, black) glucose concentration for 12 h (n = 3, each group). Relative pJNK and pAkt expressions were determined by densitometry, and they were normalized using total JNK and Akt levels. Representative images were selected from 3 independent experiments; n = 3 in each group; data are expressed as mean ± SEM; *P<0.05, between the indicated groups.

Next, we assessed the effects of high-glucose treatment on JNK phosphorylation (**Fig 5A**). Phosphorylation of JNK after 12 h of high-glucose treatment was shown to be significantly increased compared with the levels observed at regular glucose conditions. However, Akt phosphorylation after 12 h of high-glucose treatment was significantly suppressed (**Fig 5B**). The phosphorylation of another stress-activated kinase, p38, was shown to be unaffected by high-glucose treatment (**S1 Fig**). To assess the possible effects of osmolality caused by high-glucose treatment, we treated cells with non-metabolic glucose analogue 2-deoxyglucose (2-DG). The addition of 13.9 mM 2-DG to 11.1 mM glucose-containing RPMI medium, in order to adjust the osmolality to 25 mM glucose-containing medium, did not affect the phosphorylation of JNK, whereas 25 mM high-glucose treatment significantly increased JNK phosphorylation (**Fig 5C**).

### JNK signaling inhibition suppresses abnormal glucagon secretion induced by high-glucose treatment

**Fig 6:**
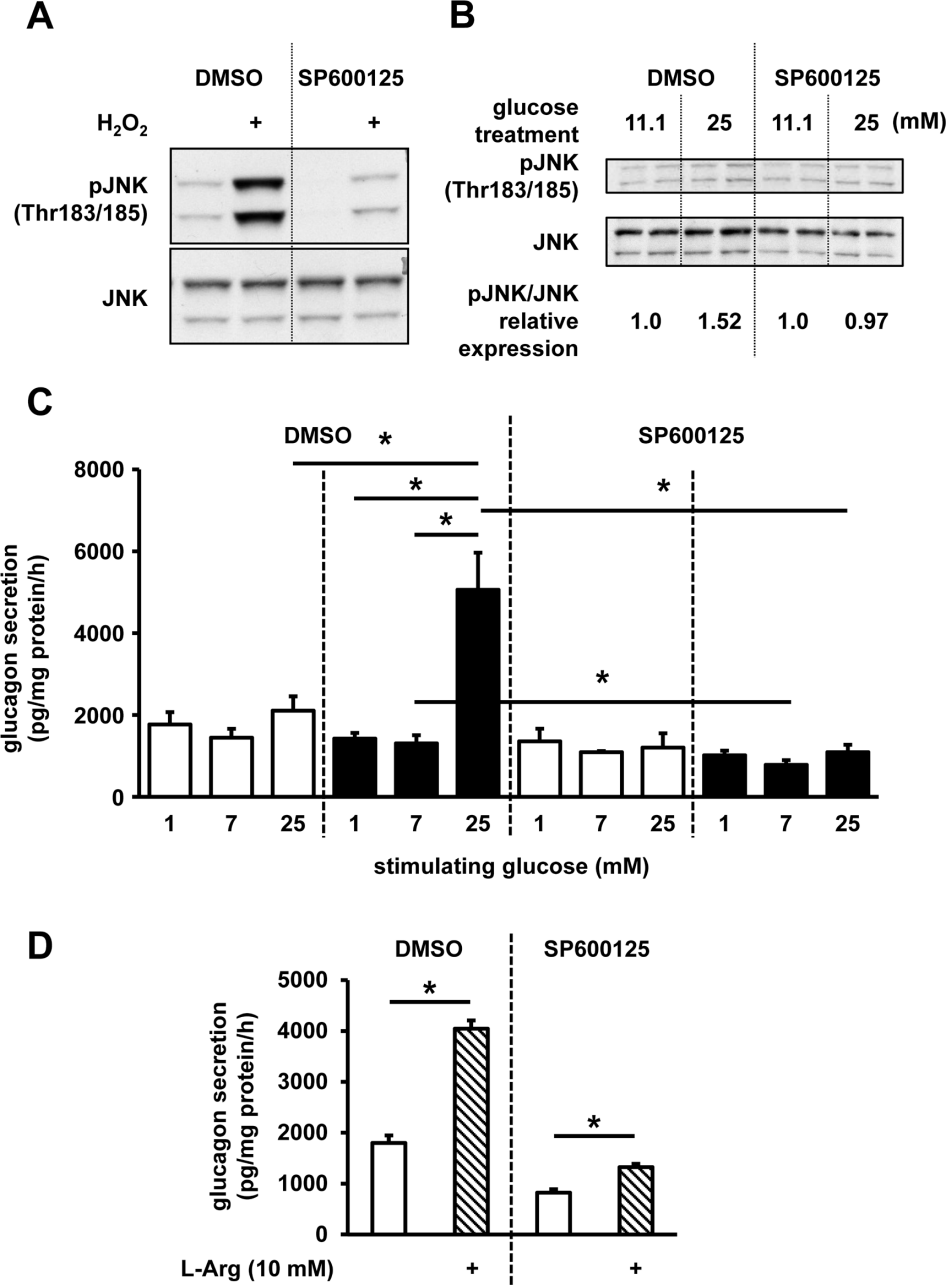
Effects of JNK inhibition on InR1G glucagon secretion. (**A**) Cells were cultured with DMSO or 10 μM SP600125 for 1 h, and stimulated using H_2_O_2_ (100 μM) for 15 min, and pJNK and JNK levels were determined. (**B**) JNK and pJNK levels in cells treated with regular (11.1 mM) or high (25 mM) glucose concentrations for 12 h, with the addition of DMSO or 10 μM SP600125. Relative pJNK expressions were determined by densitometry, and they were normalized using total JNK levels. (**C**) Glucagon secretion in cells treated with regular (11.1 mM, white) or high (25 mM, black) glucose concentrations, together with DMSO or 10 μM SP600125 for 12 h before the static incubation with the indicated glucose levels. (**D**) Cells were treated with DMSO or 10 μM SP600125, for 12 h before the static incubation with 7 mM glucose with (hatched)/without the addition of 10 mM L-Arg. Representative graphs/images selected from the results of 3 or 4 independent experiments are shown. n = 3–4 in each group; data are expressed as mean ± SEM; *P<0.05, between the indicated groups.

To explore the role of JNK signaling in the dysregulated glucagon secretion, JNK signaling was inhibited by SP600125, a specific inhibitor, and we assessed the changes in glucagon secretion. The suppression of JNK phosphorylation was confirmed in hydrogen peroxide-treated InR1G cells (100 μM, 15 min; **Fig 6A**). Additionally, SP600125 was shown to prevent the upregulation of JNK phosphorylation following the high-glucose treatment (**Fig 6B**). Next, we demonstrated that 12-h treatment of these cells using this inhibitor suppresses the high-glucose treatment-induced upregulation of glucagon secretion, and glucagon levels return to the level observed under the regular culture condition (**Fig 6C**). The cells were also treated with SP600125, and afterward stimulated with L-Arg, known to strongly stimulate glucagon secretion, independently of glucose. Following the SP600125 treatment, glucagon secretion was shown to remain significantly stimulated by L-Arg, although the glucagon levels were decreased (**Fig 6D**). In contrast, treatment of InR1G cells with an Akt-specific inhibitor A6730 did not lead to a significant change in glucagon secretion levels (**S2 Fig**).

**Fig 7:**
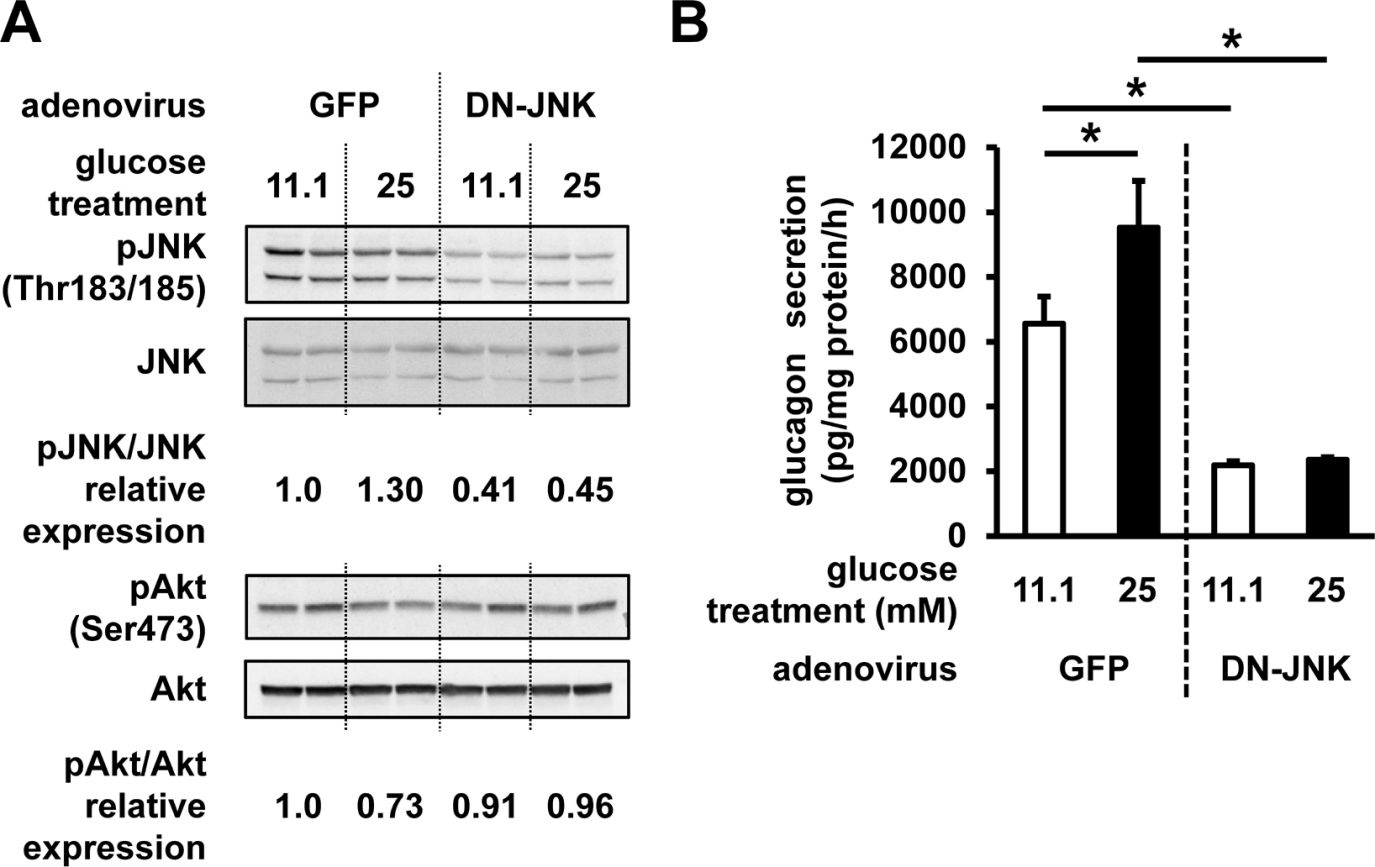
Effects of DN-JNK expression on InR1G glucagon secretion. Cells were infected with adenoviruses expressing GFP or DN-JNK 24 h prior to the 12-h treatment with 11.1 or 25 mM of glucose. (**A**) pJNK, JNK, pAkt, and Akt expression levels in these cells. Relative pJNK and pAkt expressions were determined by densitometry, and they were normalized using total JNK and Akt levels. (**B**) Glucagon secretion was assessed at 25 mM glucose stimulation in cells infected with adenoviruses carrying GFP- or DN-JNK expression vector. n = 4~6 in each group; data are expressed as mean ± SEM; *P<0.05, between the indicated groups.

To further explore the role of JNK and Akt signaling on the glucagon secretion, JNK signaling was inhibited by the ectopic expression of DN-JNK using the adenoviral gene transfer. DN-JNK overexpression was shown to reduce the phosphorylation of JNK, and to ameliorate the impaired phosphorylation of Akt (**Fig 7A**). Glucagon secretion by cells infected with the control GFP-expressing vector was shown to be elevated after regular (11.1 mM) glucose treatment, most likely due to the virus infection (**Fig 7B**). Glucagon secretion was significantly increased after the high (25 mM) glucose treatment as well, in comparison with that measured following the treatment with the regular concentrations of glucose. In contrast to this, DN-JNK expression led to a decrease in glucagon secretion and high-glucose treatment-induced upregulation of glucagon secretion (**Fig 7B**).

## Discussion

The mechanisms underlying dysregulated glucagon secretion in diabetes, both physiological and molecular, are still unclear. In this study, we succeeded in the reproduction of abnormally elevated glucagon secretion, similar to the diabetic phenotype, *in vitro*. Additionally, the obtained data indicate a possible pathophysiological process leading to the abnormal glucagon secretion under diabetic conditions: oxidative stress-induced dysregulation of insulin signaling. Insulin signaling in α-cells represents one of the critical mechanisms for the regulation of physiological glucagon secretion [7]. In diabetic state, however, the signaling is known to be impaired in various tissues, including liver and adipose tissue [25]. This indicates that the deterioration of insulin signaling in pancreatic α-cells in diabetes can play certain roles in the dysregulation of glucagon secretion similar to that in the classical target organs for insulin activity.

To date, several mechanisms were suggested to be responsible for the dysregulated glucagon secretion in diabetes [17]. These include nervous system dysfunctions, impairment of endocrine regulation, and the dysregulation of nutrient sensing in the α-cells. Here, we analyzed one of the cell-autonomous mechanisms involved in the dysregulation of glucagon secretion. The deterioration of this cellular endogenous mechanism may disturb the interactions between islet cells. The intra-islet regulation of α-cells by the surrounding islet cells, such as β- and δ-cells, was recently identified as pivotal for the fine-tuning of physiological glucagon secretion [7]. As demonstrated here, the deterioration of insulin signaling in α-cells in diabetic hyperglycemia may exacerbate the intra-islet insulin effects on α-cells and may trigger the characteristic dysregulation of glucagon secretion. The insulin resistance of α-cells was proposed as a pathophysiological mechanism, in addition to classical insulin targets, such as liver, skeletal muscles, and adipose tissue [25].

Surprisingly, the abnormally elevated glucagon secretion was easily induced by exposing isolated islets and InR1G cells to high glucose levels, which is similar to abnormal glucagon secretion observed in diabetes [26, 27]. Glucotoxicity has become a widely accepted concept as one of the main mechanisms involved in the development of diabetic disorder. The significance of glucotoxicity in β-cells, leading to the deterioration of cellular function, including insulin secretion, is especially recognized and intensively investigated. Oxidative stress is one of the important factors inducing β-cell glucotoxicity in the conditions of diabetic hyperglycemia [28]. A variety of cellular stresses, including oxidative stress, impairs insulin signaling through the alterations of JNK pathway [28]. JNK pathway was reported to suppress insulin signaling, including Akt activity, through the serine phosphorylation of insulin receptor substrate 1 (IRS-1), which further inhibits tyrosine phosphorylation [29]. We showed that the high-glucose treatment induces the upregulation of ROS in InR1G cells, which is followed by JNK activation. Among different stimuli, high osmolality induced by the exposure to high glucose levels was shown to activate this pathway [30, 31]. We assessed the effects of high osmolality on InR1G cells, and the addition of 2-DG did not activate JNK pathway, indicating that high glucose, which can be metabolized by the target cells and induce oxidative stress, specifically upregulates JNK phosphorylation [28]. Phosphorylation of JNK was significantly increased after 12 h of high-glucose treatment. In the relatively short-term treatment points, the phospho-JNK levels were increasing, but this was not statistically significant when compared with the other treatment points. Given that a longer period of exposure to high-glucose levels is necessary to induce certain oxidative stress in the cells [28], this increasing tendency of phospho-JNK levels in short-term high-glucose treatment could be due to environmental changes, including cultural medium change or serum starvation, which can also evoke certain stresses to the cells. Excessive glucose levels may induce endoplasmic reticulum (ER) stress as well, which represents a well-known upstream mechanism that induces JNK signaling [32], and ER stress in α-cells was reported to induce the alterations in insulin signaling and dysfunctional glucagon secretion by affecting JNK signaling [33]. The results obtained here show that the inhibition of JNK and the expression of DN-JNK suppress glucagon secretion, demonstrating the pivotal role of JNK in the regulation of glucagon secretion.

Furthermore, we showed that glucagon content in cells significantly decreases after 12 h of high-glucose treatment. In contrast to this, preproglucagon expression tended to increase in these cells. Previous studies demonstrated that the extended high-glucose treatment of α-cells induces upregulation of glucagon expression [24, 34]. However, the observed slight change in gene expression in this study may be explained by a shorter period of high-glucose treatment, in comparison with the previous studies, where the cells were exposed to high glucose for 5 days [24]. Indeed, extended high-glucose treatment of InR1G cells for 120 h induced significant upregulation of proglucagon expression in this study. We demonstrated that 12-h high-glucose treatment induced the increase in glucagon secretion, which may suggest that 12-h high-glucose stimulation can induce a continuous increase in glucagon secretion, but the *de novo* biosynthesis of glucagon requires more time. Indeed, it was reported that an 8-h treatment of αTC1 cells led to an increase in cumulative glucagon levels in the culture medium [35]. Therefore, the decrease in the cellular glucagon content after 12 h of high-glucose treatment may mirror the continuous increase in glucagon release during the treatment period. Compared with the gene expression changes, signaling-pathway changes are much quicker, and therefore, environmental stimuli can induce the changes in these pathways. In the pre-diabetic state, repeating periods of hyperglycemia, especially in postprandial state, can induce the changes in α-cell signaling pathways. These relatively faster changes may be preceding chronic hyperglucagonemia observed in diabetic state, and partially initiate hyperglycemia at the early stage of the disease [3].

Here, we propose glucotoxicity as one of the mechanisms underlying glucagon dysregulation in α-cells, commonly observed under diabetic conditions. In diabetic patients, the reconstitution of adequate blood glucose levels during oral glucose load test, using an artificial endocrine pancreas, normalized abnormally elevated glucagon secretion [36]. Additionally, insufficient glucagon response to hypoglycemia in diabetic patients was shown to be normalized by the maintenance of long-term (1 to 3 months) adequate glycemic control using intensive insulin therapy [37]. These findings indicate the involvement of glucotoxicity in abnormal glucagon secretion, which is reversible by adequate glycemic control.

The limitation of our study is the use of an *in vitro* model instead of *in vivo* studies. However, *in vivo* glucagon and α-cell studies are limited by the accessibility and availability of α-cells and glucagon assays, which is why we used the *in vitro* model. However, as the cell lines are mostly immortalized by using gene modifications, their physiological status differs from that *in vivo*, and generally, *ex vivo* islets are used as a model for islet research [38]. InR1G cells were used in many studies, because these cells secrete glucose-induced glucagon, similar to the isolated islets [20]. Most of the islet cells are β-cells, especially in rodent models, so using these islets as a model for β-cells is justified; however, the number of α-cells is much lower. Additionally, it is known that α-cell functions, including glucagon secretion, are influenced by the islet non-α-cells via insulin produced by β-cells [39–43] and somatostatin produced by δ-cells [44]. Indeed, insulin secretion also significantly increased by high-glucose treatment (**Fig 1B**). Therefore, it was difficult to further analyze the α-cell-specific molecular mechanisms in the isolated islets. In this study, we focused on the cell-autonomous mechanisms, which do not depend on other regulatory factors. Our *in vitro* model allowed the assessment of cellular intrinsic responses and mechanisms disregarding other, environmental influences, including the effect of insulin produced by the surrounding β-cells *in vivo*. Glucagon secretion dynamics in the isolated cells was reported to be different from that in the cells under *in vivo* physiological conditions [45], but we believe that our findings may provide a crucial understanding of the cellular mechanism of glucagon regulation. The results obtained in our experiments are somewhat different from those reported in the previous studies [20], which may be due to the differences in the experimental conditions, and particularly, due to the different assays for the determination of glucagon concentration used in our and previous studies. In one previous report [20], glucagon concentration in the experimental buffer was evaluated by a conventional radioimmunoassay (RIA) system, unlike glucagon-specific sandwich enzyme-linked immunosorbent assay (ELISA) used here. Recently, the specificity of conventional RIA for the determination of glucagon content was questioned due to the specificity of used anti-glucagon antibody [46], and, therefore, it is possible that the change in glucagon secretion was overestimated in some previous reports. Additionally, the analysis of glucagon secretion from InR1G cells is not affected by the presence of intestinal proglucagon-derived peptides and potential cross-reactivity with other molecules. However, the mechanisms described in this work should be validated using improved model systems.

## Conclusions

Here, we explored the molecular mechanisms underlying glucagon dysregulation, and demonstrated that the impaired insulin signaling can be induced by high glucose load, leading to oxidative stress and the upregulation of JNK signaling in glucagon-secreting InR1G cells. To date, little is known about the dysregulation of glucagon secretion under diabetic conditions, even though this is crucial for the development of novel treatment strategies. Therefore, the results obtained here provide an insight into the mechanism of abnormal glucagon secretion, and contribute to the understanding of diabetes through glucagon pathophysiology.

## Supporting information

S1 Fig Phosphorylation status of p38. InR1G cells were exposed to regular (11.1 mM, white) or high (25 mM, black) glucose levels for 12 h. (**A**) Phospho-(p)p38 and p38 levels. (**B**) Relative expression of p-p38 was determined using densitometry and normalized using total p38 levels. n = 3 in each group. Data are expressed as mean ± SEM.
(PDF)

S2 Fig Effects of Akt inhibition on InR1G glucagon secretion. Cells were cultured with A6730 (Sigma-Aldrich, U.S.A.) in indicated concentrations for 12 h. (**A**) Akt and pAkt levels. (**B**) Glucagon secretion in cells treated without (white) or with 1 μM A6730 (gray) for 12 h before the static incubation with 25 mM glucose. n = 3–4 in each group; data are expressed as mean ± SEM.
(PDF)
