# Supplementary material for: Glucotoxicity induces abnormal glucagon secretion through impaired insulin signaling in InR1G cells
Source: PLoS One. 2017 Apr 20;12(4):e0176271. doi: 10.1371/journal.pone.0176271 (PMC5398759; doi:10.1371/journal.pone.0176271)
Supplement: S1 Fig — InR1G cells were exposed to regular (11.1 mM, white) or high (25 mM, black) glucose levels for 12 h. (A) Phospho-(p)p38 and p38 levels. (B) Relative expression of p-p38 was determined using densitometry and normalized using total p38 levels. n = 3 in each group. Data are expressed as mean ± SEM. (PDF) [file pone.0176271.s001.pdf]

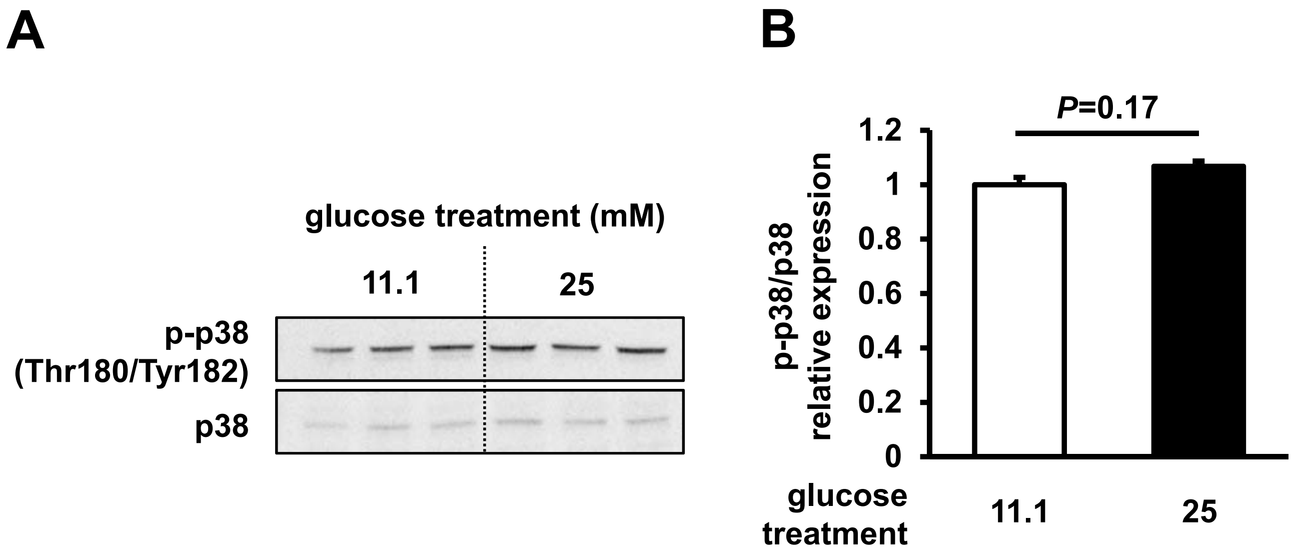

S1 Figure. Phosphorylation status of p38. InR1G cells were exposed to regular (11.1 mM, white) or high (25 mM, black) glucose levels for 12 h. (**A**) Phospho-(p)p38 and p38 levels. (**B**) Relative expression of p-p38 was determined using densitometry and normalized using total p38 levels. n=3 in each group. Data are expressed as mean ± SEM.
