# Supplementary material for: Glucotoxicity induces abnormal glucagon secretion through impaired insulin signaling in InR1G cells
Source: PLoS One. 2017 Apr 20;12(4):e0176271. doi: 10.1371/journal.pone.0176271 (PMC5398759; doi:10.1371/journal.pone.0176271)
Supplement: S2 Fig — Cells were cultured with A6730 (Sigma-Aldrich, U.S.A.) in indicated concentrations for 12 h. (A) Akt and pAkt levels. (B) Glucagon secretion in cells treated without (white) or with 1 μM A6730 (gray) for 12 h before the static incubation with 25 mM glucose. n = 3–4 in each group; data are expressed as mean ± SEM. (PDF) [file pone.0176271.s002.pdf]

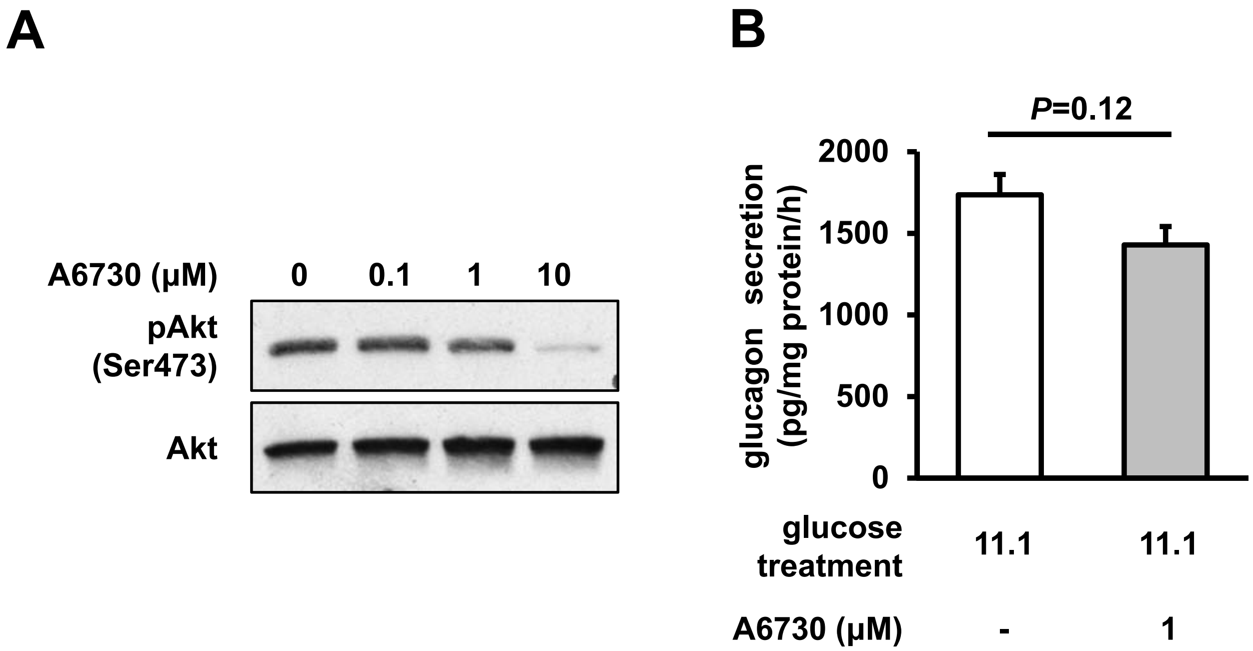

S2 Figure. Effects of Akt inhibition on InR1G glucagon secretion. Cells were cultured with A6730 (Sigma-Aldrich, U.S.A.) in indicated concentrations for 12 h. (**A**) Akt and pAkt levels. (**B**) Glucagon secretion in cells treated without (white) or with 1 μM A6730 (gray) for 12 h before the static incubation with 25 mM glucose. n=3-4 in each group; data are expressed as mean ± SEM.
